# Managing emotional distress in older adults with mental illness: a randomized controlled trial evaluating virtual reality relaxation

**DOI:** 10.1038/s41398-026-03955-1

**Published:** 2026-03-20

**Authors:** M. Seethaler, L. Güntner, A. Lütt, S. A. Just

**Affiliations:** 1 Charité – Universitätsmedizin Berlin, Department of Psychiatry and Psychotherapy, Campus Mitte, Berlin, Germany; 2 Department of Psychiatry, Charité University Hospital at St. Hedwig Hospital, Berlin, Germany; 3 Berlin Institute of Health at Charité – Universitätsmedizin Berlin, Berlin, Germany; 4 German Center for Mental Health (DZPG), partner site Berlin, Berlin, Germany; 5 Department of Education and Psychology, Freie Universität Berlin, Berlin, Germany; 6 Department of Clinical Medicine, UiT – The Arctic University of Norway, Tromsø, Norway

**Keywords:** Psychiatric disorders, Scientific community

## Abstract

Virtual reality (VR) relaxation offers an innovative, immersive approach to managing negative emotions. Such digital therapies represent a promising, growing field in mental health care but remain under-researched in older populations – a group in critical need of scalable, engaging treatments. We conducted a randomized controlled trial to evaluate the feasibility and effectiveness of VR-based relaxation compared to guided imagery (treatment-as-usual) in older adults with mental illness. 44 older psychiatric patients (aged 58–98) were randomized into either VR or guided imagery (GI). In total, 39 participants completed the study (VR: n = 21, GI: n = 18) and were selected for analysis. Dropout rates, satisfaction, and overall experience were indicators of feasibility in both groups. Additionally, motion sickness and sense of presence were assessed in the VR group. Effectiveness was evaluated through pre-post-measurements of state anxiety (STAI-X1), affect (PANAS), and visual analogue scales for stress, relaxation, and well-being. VR demonstrated strong feasibility, with low dropout rates, high satisfaction and immersion, and minimal motion sickness (M = 0.25 ± 0.91). Mixed repeated measures ANOVA analyses revealed significant improvements in state anxiety, stress, negative affect, relaxation, positive affect, and well-being across both groups (p < 0.001), with no significant differences between VR and GI. Feasibility and effectiveness were consistent across age, gender and severity of illness. Our findings suggest that VR relaxation is a feasible and effective intervention for older adults with mental illness, offering a comparable alternative to traditional relaxation methods. This study underscores the potential of VR to enhance mental health care for older populations, including those with advanced age and serious mental illness.

## Introduction

The ‘digital gray divide’ in mental health refers to the reduced investigation and use of advanced technologies such as virtual reality (VR) in the treatment of older adults with mental illness [1–3]. To ensure that digital interventions reach and benefit this population, systematic evaluations are warranted [4]. When applied ethically, advanced technologies hold the potential to address critical challenges faced by older adults and significantly enhance their quality of life. We therefore present results from a randomized controlled trial (RCT) comparing the feasibility and effectiveness of VR-based relaxation to more traditional methods in older adults with mental illness.

Conventional relaxation techniques aim to alleviate stress and anxiety by triggering physical calming responses, including slower respiration and reduced heart rate [5, 6]. A commonly employed method is guided imagery (GI), which involves mentally visualizing serene scenarios – such as strolling through a forest – often accompanied by an audio guide [7, 8]. In psychiatric settings, GI is frequently integrated as a supportive technique to help manage emotional distress across a range of mental health disorders [9, 10].

Evidence suggests that GI can reduce both mental and physical symptoms. For example, participants with depression reported fewer depressive symptoms, anxiety, and stress after GI sessions [11]. Its effectiveness in alleviating anxiety, depression, pain, and fatigue in palliative care was described in a systematic review [12]. Among older adults, GI has shown benefits for stress, sleep quality, and health-related quality of life [13–15]. Despite these advantages, GI’s non-immersive nature requires strong mental imagery skills and sustained attention, which can be challenging for older patients [16].

VR offers an alternative to GI, enabling immersive, interactive environments without external distractions [17–19]. Unlike GI, VR can place users directly into a virtual calming space, enhancing presence, as “being there” in this virtual environment, and focus [20, 21]. VR guided meditation has been perceived as more immersive and conducive to concentration [16]. This may be particularly useful for older adults with psychiatric or neurological conditions, where anxiety and attention deficits are common [22, 23].

Research on VR relaxation in psychiatric contexts is growing but remains limited in older populations. Prior studies have explored its application e.g. in mood, anxiety, psychotic, and substance use disorders, primarily among younger adults [24–27]. A systematic review by Riches et al. found that VR relaxation was equally or more effective than non-virtual methods in reducing stress and anxiety in psychiatric patients [9].

In one study, VR led to significantly lower negative affect compared to audio-guided relaxation in psychiatric outpatients [10]. Physiological markers of relaxation, such as decreased skin conductance and slower respiration, were found in patients with substance use disorders following VR exposure [25]. For patients with generalized anxiety disorder, VR yielded improvements in physiological anxiety markers and higher completion rates compared to GI or a passive control group [28, 29].

Despite these promising results, most VR studies have focused on younger adults and outpatients, with limiting generalizability to older, clinically complex populations [9]. In geriatric psychiatry, VR research has mostly targeted cognitive impairments, with little attention to relaxation interventions [30, 31]. Meanwhile, VR has shown feasibility in older adults with psychiatric conditions, with patients aged 58–92 reporting strong presence and well-being during VR use, with low dropout rates and minimal motion sickness [32].

To close the ‘digital gray divide’ in mental health and ensure equitable access to effective treatments for all age groups, more systematic investigations of advanced, promising technologies such as VR are critical – particularly in light of an aging population and the growing demand for innovative psychiatric care [4, 33–35].

Therefore, the present RCT examined the effectiveness and feasibility of VR-based versus GI-based relaxation interventions in geriatric psychiatry. As therapeutic interventions in this setting are typically transdiagnostic, we included older patients with diverse psychiatric conditions to reflect routine clinical practice. We hypothesized that both interventions would reduce state anxiety to a similar extent, negative affect, and perceived stress, while increasing well-being, positive affect, and relaxation. Additionally, we explored whether VR would yield greater improvements in these outcomes and whether a higher sense of presence in VR would predict lower post-intervention anxiety. Feasibility was assessed through dropout rates, user satisfaction, and experience. We also hypothesized that VR would be well-tolerated, with low motion sickness and no influence of demographic or clinical factors on intervention feasibility.

## Methods

### Participants

**Fig. 1:**
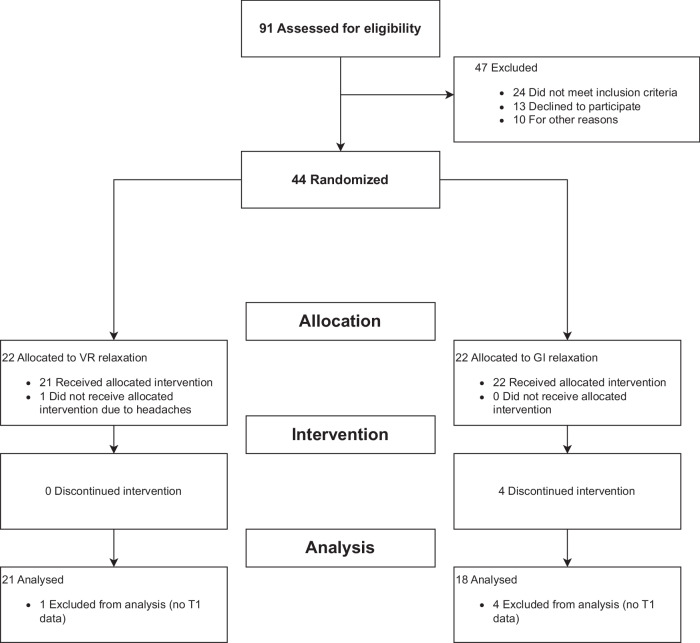
CONSORT flow diagram. 91 patients were assessed for eligibility. After exclusion of 47 patients (24 not meeting inclusion criteria, 13 declining to participate, 10 being excluded due to other reasons), 44 participants were included and randomized. 22 participants each were allocated to both the VR and the GI intervention. Of these, 22 received the GI relaxation, and 21 received the VR relaxation with 1 person declining to receive the intervention due to headaches. 4 participants of the GI group and none of the VR group discontinued the intervention. 18 participants of the GI group and 21 participants of the VR group were analyzed.

Due to the lack of RCTs on VR-based relaxation in mental illness – and none in geriatric psychiatry – a precise a priori sample size calculation was not feasible. Based on comparable sample sizes of previous studies investigating VR relaxation versus standard relaxation or a passive control condition in mental illnesses [10, 25, 28, 36], we aimed to recruit 20 participants per group. A total of N = 44 patients (59% females) aged 58 – 87 years (M = 70.98, SD = 7.42) were recruited from the inpatient, day clinic, and outpatient department at the Psychiatric University Clinic of the Charité at St. Hedwig Hospital Berlin, Germany. The CONSORT Flowchart of the RCT is shown in Fig. 1, sample characteristics are presented in Table 1. Data from n = 39 were included in the final analysis; four participants dropped out from the GI group, none from VR, although one person did not receive the allocated VR intervention due to headaches.

**Table 1:** Characteristics of sample at baseline (N = 44).

	VR (n = 22)	GI (n = 22)	p-value
Age	71.41 ( ± 6.95) 62.00 – 87.00	70.55 ( ± 8.01) 58.00 – 84.00	0.704
Gender (female)	14 (63.64%)	12 (54.55%)	0.540
Education years	13.84 ( ± 3.65) 4.00 – 20.00	13.00 ( ± 2.49) 8.00 – 18.00	0.377
Main psychiatric diagnosis			1.000
Organic mental disorders (F0x)	2 (9.09%)	3 (13.64%)	
Substance use disorders (F1x)	3 (13.64%)	3 (13.64%)	
Schizophrenia, schizotypal & delusional disorders (F2x)	1 (4.55%)	1 (4.55%)	
Mood disorders (F3x)	12 (54.55%)	11 (50.00%)	
Neurotic, stress-related & somatoform disorders (F4x)	4 (18.18%)	4 (18.18%)	
Psychiatric comorbidity	16 (72.73%)	11 (50.00%)	0.122
Somatic comorbidity	16 (72.73%)	15 (68.18%)	0.741
Setting			0.753
Inpatient	6 (27.27%)	4 (18.18%)	
Day clinic	11(50.00%)	13 (59.09%)	
Outpatient	5 (22.73%)	5 (22.73%)	
Care level			0.025*
0 = none	18 (81.82%)	9 (40.91%)	
1 = minor	1 (4.55%)	6 (27.27%)	
2 = significant	3 (13.64%)	5 (22.73%)	
3 = severe	0 (0.00%)	2 (9.09%)	
4/5 = most severe	0 (0.00%)	0 (0.00%)	

Categorical variables: absolute and relative frequencies (n, %) are reported as well as p-values from X² tests or Fisher’s exact tests for group comparisons; continuous variables: means, standard deviations (M ± SD) and ranges are reported as well as p-values from independent t-tests for group comparisons. Comorbid psychiatric and somatic conditions refer to the presence of any additional diagnosed mental or physical disorders. Primary psychiatric diagnoses were coded based on ICD-10 [39].

*p < 0.05.

Inclusion and exclusion criteria followed the study by Just & Lütt et al. on VR feasibility in geriatric psychiatry [32]. Patients with various psychiatric diagnoses were included if they had sufficient German language skills, were able to understand the procedure, and provided informed written consent. Exclusion criteria were the inability to provide informed written consent or the physical inability to take part in the study – which included patients with severe dementia or delirium, acute substance use disorders, and severe neurological conditions. Patients with acute suicidality and aggressive behavior were also excluded. Furthermore, patients were not eligible to participate during the first week following a transfer or change in setting. Inclusion criteria and consent capacity were confirmed by the treating clinicians.

### Study design and procedure

A RCT with two relaxing conditions was conducted: (1) VR-based relaxation intervention and (2) GI-based dream journey (treatment as usual) with two factors being varied: time (within-subjects) and group (between-subjects). Each intervention was delivered in a single session. Given the immediate benefits of even brief relaxation sessions and limited evidence in geriatric psychiatry [9, 16, 25], we chose this design to assess feasibility and initial effects while minimizing patient risk and burden. Participants were randomly allocated 1:1 to both conditions by block randomization [37]. The group assignment was statistically generated, with allocations randomly permuted within each block, minimizing the assessor’s influence. We used a block size of 4, as recommended for two treatment conditions [38].

The second author guided through and was present during the entire process. While receiving detailed information on the purpose, content, and duration of the study as well as on the random assignment to one of two separate conditions, the participants were not informed about the type of relaxation exercise they were about to receive. The participants completed an interview on sociodemographic and medical data followed by self-report questionnaires for baseline measurement (T0) according to their allocated condition.

*VR group:* Participants received a brief introduction on using the Oculus Meta Quest 2 head-mounted display (Reality Labs, Meta Platforms Inc) and navigating the virtual environment. The 10-minute relaxation scenario, launched via the app *Nature Treks VR* (Greenergames, released on May 21, 2019), featured a forest landscape with a river, mountains, animals, and calming nature sounds. All participants experienced the same virtual setting to ensure standardization across sessions.

*GI group:* Participants in the GI group received an audio-guided imagery exercise delivered by the investigator. The 10-minute dream journey was developed specifically for this study and mirrored the VR environment in both duration and content, describing the forest scene illustrated above (Appendix A). To ensure comparability, only elements also present in the VR setting were included.

After the intervention, participants of the VR group rated their current level of motion sickness and both groups completed the post-treatment (T1) self-report questionnaires according to their assigned condition.

### Measures

A mixed-methods design was used to assess effectiveness, feasibility, and sample characteristics. Effectiveness outcomes – state anxiety, affective states, stress, relaxation, and well-being – were measured pre- (T0) and post-intervention (T1), and selected based on prior VR research [9]. Feasibility was evaluated at T1 alongside dropout rates, satisfaction, and user experience. Sense of presence and motion sickness were only recorded in the VR group. Both the T0 and T1 assessment were conducted within the same session, immediately before and after each relaxation intervention.

*Sociodemographic and clinical data* were extracted from electronic health records and a brief pre-intervention interview. Variables included age, diagnosis (ICD-10; [39]), comorbidities, care level, education, and prior experience and attitude toward relaxation techniques. The VR group also answered additional questions regarding their familiarity with and perception of VR.

*Feasibility* was investigated through dropout rates as well as additional measures: Dropouts were defined as early termination of the intervention, with timing and reasons documented. Patient satisfaction was measured post-intervention using five adapted items from the ZUF-8 questionnaire, assessing overall satisfaction, how well the intervention met patient needs, perceived quality, and willingness to repeat or recommend it [40]. In line with prior research [41, 42], we set a cut-off score of >12.5 (out of 20) to indicate acceptable satisfaction. Experience of the relaxing environment was measured with three single-item visual analogue scales (VAS), ranging from 0 = ‘not at all’ to 10 = ‘very’, that assessed helpfulness, connectedness to nature, and immersion. To measure immersion, participants were asked to what extent they felt that they actually were in the virtual or imagined environment. The VAS were adapted from a study by Riches et al. [43]. In the VR group, motion sickness was assessed post-treatment using the Fast Motion Sickness Scale (FMS; [44]), asking participants to rate their motion sickness on a scale from 0 = ‘no motion sickness’ to 20 = ‘frank motion sickness’. Sense of presence in the VR group was measured using the German version of the iGroup Presence Questionnaire (IPQ; [45]). The 14 IPQ items are divided into the subscales ‘spatial presence’, ‘involvement’, and ‘realness’, with one additional item measuring ‘general presence’. Mean scores were calculated for the entire scale, with a higher overall score indicating a higher sense of presence.

*Effectiveness* was assessed as follows: State anxiety was measured using the German version of the STAI-X1 [46, 47]. Positive and negative affect were assessed with the German PANAS [48–50]. Perceived stress, relaxation, and well-being were each measured using single-item visual analogue scales (VAS), ranging from 0–10 with lower scores indicating less stress, as well as higher scores reflecting greater relaxation and well-being. The items were based on previous studies examining the effects of VR relaxation in individuals with mental illness [36, 43].

### Statistics

All analyses were conducted using RStudio (R 5.1), figures were created with Python 3.11. The significance level was set at p < 0.05. Single missing values were imputed using the mean of the corresponding scale or subscale. There were no cases where more than one value was missing from a scale. Repeating analyses without cases with missing values did not change any results. Outliers were identified via boxplots and retained unless due to measurement error.

Dropout rates and participant characteristics were analyzed using all available data (N = 44). For other analyses, only cases with both pre- and post-measurements were included (n = 39). A mixed repeated measures ANOVA examined changes in effectiveness outcomes within and between groups. Assumptions were tested and met (homogeneity of variance, covariance, sphericity, independence, and residual normality), except for slight deviations from normality in stress and negative affect. Therefore, cluster bootstrapping (1000 resamples) was used for robust estimation of effect sizes and confidence intervals. FDR Benjamini-Hochberg correction adjusted for multiple comparisons.

Multiple regression analysis in the VR group tested whether presence and baseline anxiety predicted post-treatment anxiety. Variables were z-standardized for the analysis. Assumptions (linearity, normality, homoscedasticity, multicollinearity, and independence) were met. Potential outliers (Cook’s Distance < 0.5) did not impact results and were retained.

Descriptive statistics summarized feasibility outcomes. For non-normally distributed variables, medians and interquartile ranges (IQR) were reported. Group differences were analyzed using appropriate non-parametric or categorical tests (Fisher’s exact, Spearman, Mann-Whitney U, Kruskal-Wallis).

## Results

There were no significant group differences in baseline demographic or clinical characteristics, except for care level (Fisher’s exact test: p = 0.025): twice as many patients in the VR group were not dependent on care, while the GI group contained more patients with a minor or significant care dependency. Most participants in both groups had prior experience with traditional relaxation techniques (VR: n = 18; GI: n = 19). In the VR group, only three patients had previously used any kind of VR. The attitude toward conventional relaxation methods was positive across all participants, with an average value of 4.11 (SD = 1.17) on a Likert scale where 5 presented the most positive value. Participants in the VR group showed a neutral attitude toward VR (M = 3.18, SD = 1.26).

### Feasibility

Five participants discontinued the study prematurely. In the VR group, one participant withdrew prior to the intervention due to a headache, and was therefore excluded from dropout analyses. While nobody dropped out in the VR group, four GI participants terminated the session prematurely, citing increased anxiety, distressing memories, irritability/concentration difficulties or limited imaginative capacity during relaxation. This difference in dropout rates was not statistically significant (Fisher’s exact test: p = 0.108).

Patient satisfaction, measured by five adapted ZUF-8 items, yielded median scores above the pre-defined threshold of >12.5 in both groups (VR: Mdn = 18, IQR = 4; GI: Mdn = 17, IQR = 1.5) which did not differ significantly (U = 187.00, p = 0.966).

Post-treatment experience was assessed via three VAS items: immersion, helpfulness, and connectedness to nature. All three were significantly correlated (r = 0.34 – 0.50, p < 0.05). Median scores were high (10 represented the highest possible score) and did not differ significantly between groups for immersion (VR: Mdn = 8.3, IQR = 4.6; GI: Mdn = 7.9, IQR = 3.53, U = 194.5, p = 0.888), helpfulness (VR: Mdn = 8.8, IQR = 4.2; GI: Mdn = 8.05, IQR = 3.5, U = 181, p = 0.832), and connectedness to nature (VR: Mdn = 9.2, IQR = 2; GI: Mdn = 10, IQR = 2.08, U = 162, p = 0.436. Scattered boxplots visualizing patients’ experience and satisfaction by group are presented in Appendix B.

Data from 21 participants in the VR group were analyzed for estimating the feasibility of the VR-based relaxation intervention. Motion sickness, assessed via the FMS Scale (0–20), had a mean of M = 0.25 (SD = 0.91), with 95.24% of values ≤ 1. Sense of presence, measured by the IPQ (range: − 3– + 3), had a mean of M = 0.28 (SD = 1.37). 90.48% of VR participants indicated that they could envision VR being integrated into future clinical care.

Sensitivity analyses indicated that there were no significant associations between feasibility outcomes and sociodemographic/clinical characteristics in both groups (age, gender, education years, setting of treatment, psychiatric diagnosis, care level, and psychiatric or somatic comorbidity; detailed results in Appendix C). Similarly, Fisher’s exact tests revealed no significant differences between these characteristics and dropout status (all p > 0.05). For dropout analyses, age was categorized (> 70 vs. ≤ 70).

### Effectiveness

A descriptive overview of means and standard deviations for the six effectiveness outcome variables before and after the intervention is shown in Table 2.

**Table 2:** Overview of effectiveness outcomes.

		VR Pre	VR Post	GI Pre	GI Post
	N	M (SD)	M (SD)	M (SD)	M (SD)
State anxiety	39	4.31 (1.09)	3.06 (0.76)	4.27 (0.90)	3.16 (0.57)
Positive affect	39	2.65 (0.70)	3.31 (0.74)	2.74 (0.69)	3.27 (0.67)
Negative affect	39	1.47 (0.59)	1.12 (0.22)	1.48 (0.45)	1.14 (0.18)
Relaxation	39	5.30 (2.36)	8.70 (1.79)	5.56 (3.06)	8.82 (1.26)
Well-being	39	5.60 (2.79)	8.82 (1.62)	6.12 (2.47)	8.37 (1.86)
Stress	39	4.23 (2.72)	0.93 (1.71)	2.85 (2.37)	0.44 (0.94)

Pre/post = T0 and T1 assessments. All scales were converted to a range 0–10. State anxiety measured with STAI-X1 [46, 47], positive/negative affect measured with PANAS [49, 50], single-item VAS for stress (0 = “no stress” to 10 = “extreme stress”), relaxation (0 = “not relaxed at all” to 10 = “very relaxed”), and well-being (0 = “not well at all” to 10 = “very well”).

**Fig. 2:**
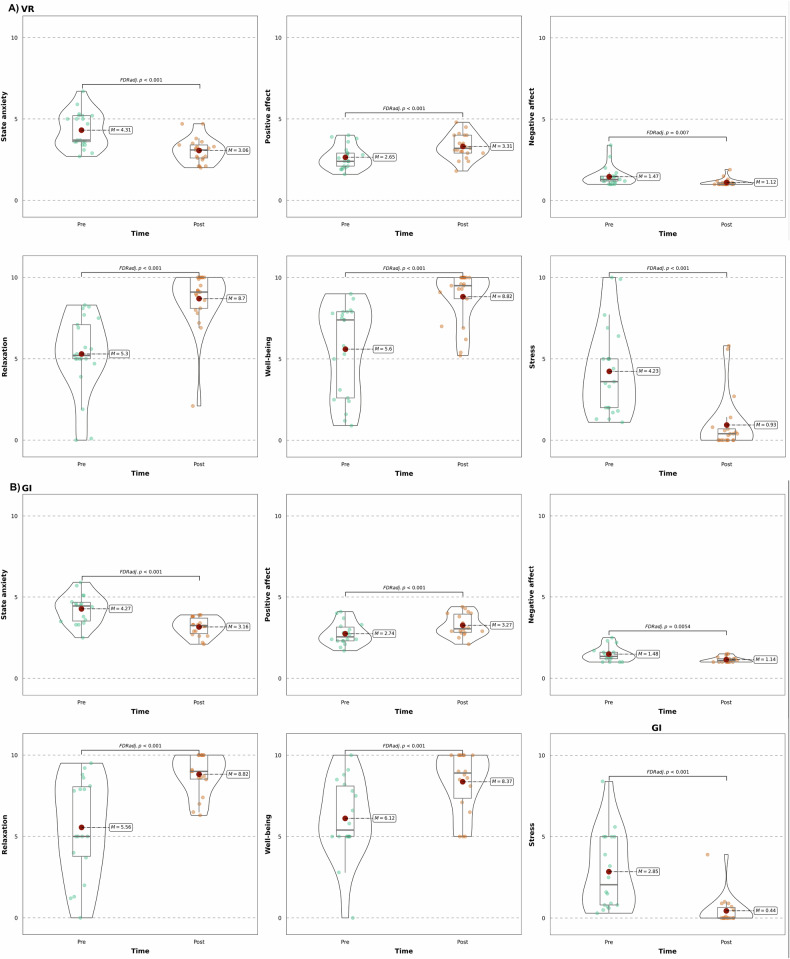
Pre-post-intervention changes in effectiveness outcomes by group. Change in state anxiety, positive and negative affect, relaxation, well-being, and stress from pre- to post-intervention by group (**A**: VR, **B**: GI). Violin plots show the density of individual scores at each time point, overlaid with the mean value and boxplots representing the median and interquartile range. All scales were converted to a range 0–10. State anxiety measured with STAI-X1 [46, 47], positive/negative affect measured with PANAS [49, 50], single-item VAS for stress (0 = “no stress” to 10 = “extreme stress”), relaxation (0 = “not relaxed at all” to 10 = “very relaxed”), and well-being (0 = = “not well at all” to 10 = “very well”). Adjusted p-values based on FDR Benjamini-Hochberg correction for 12 tests.

At baseline (T0), independent t-tests revealed no significant differences between the VR and GI groups across all outcome variables (detailed results in Appendix D). Mixed repeated measures ANOVAs showed significant main effects of time for state anxiety, positive and negative affect, relaxation, well-being, and stress (all p < 0.001), with large effect sizes (η²p = 0.34 – 0.70). No significant main effects of group or time × group interactions were observed for any outcome (all p > 0.05). Results of the mixed repeated measures ANOVA for all six outcome variables are presented in Table 3. Bootstrapped ANOVAs confirmed the robustness of the time effects (detailed results are displayed in Appendix E). Post-hoc pairwise comparisons (FDR-adjusted) revealed significant pre-post improvements in both groups and are shown in Fig. 2 (detailed results in Appendix F).

**Table 3:** Results of the mixed repeated measures ANOVA.

Outcome	Effect	F	df	η²p	95% CI for η²p
State anxiety	Group	0.02	1, 37	0.01	[0.00, 0.06]
	Time	83.52***	1, 37	0.70	[0.64, 0.78]
	Group x Time	0.28	1, 37	0.01	[0.00, 0.07]
Positive affect	Group	0.02	1, 37	0.01	[0.00, 0.07]
	Time	46.90***	1, 37	0.56	[0.49, 0.64]
	Group x Time	0.65	1, 37	0.03	[0.00, 0.10]
Negative affect	Group	0.04	1, 37	0.01	[0.00, 0.09]
	Time	18.75***	1, 37	0.34	[0.27, 0.43]
	Group x Time	0.003	1, 37	0.01	[0.00, 0.07]
Relaxation	Group	0.11	1, 37	0.01	[0.00, 0.06]
	Time	62.30***	1, 37	0.63	[0.57, 0.72]
	Group x Time	0.03	1, 37	0.01	[0.00, 0.06]
Well-being	Group	0.002	1, 37	0.01	[0.00, 0.05]
	Time	73.82***	1, 37	0.67	[0.61, 0.74]
	Group x Time	2.34	1, 37	0.07	[0.00, 0.16]
Stress	Group	2.94	1, 37	0.08	[0.01, 0.21]
	Time	56.96***	1, 37	0.61	[0.55, 0.68]
	Group x Time	1.40	1, 37	0.05	[0.00, 0.14]

η²p and 95% confidence intervals for η²p and were bootstrapped. State anxiety measured with STAI-X1 [46, 47], positive/negative affect measured with PANAS [49, 50], single-item VAS for stress (0 = “no stress” to 10 = “extreme stress”), relaxation (0 = “not relaxed at all” to 10 = “very relaxed”), and well-being (0 = “not well at all” to 10 = “very well”).

*p < 0.05, **p < 0.01, ***p < 0.001.

Sensitivity analyses showed that there were no significant associations between change in effectiveness outcomes and sociodemographic/clinical characteristics in both groups (age, gender, education years, setting of treatment, psychiatric diagnosis, care level, and psychiatric or somatic comorbidity; detailed results in Appendix G).

**Fig. 3:**
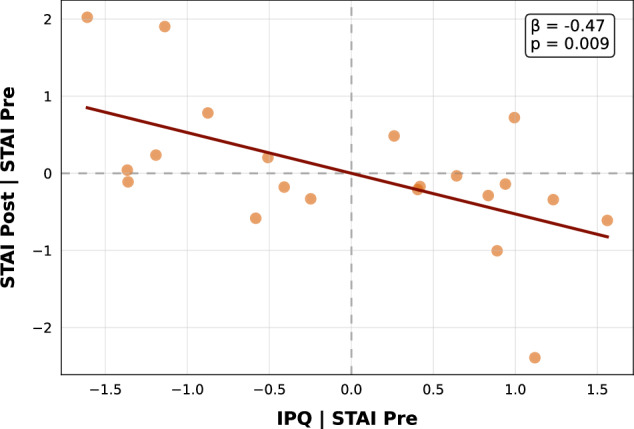
Partial regression plot of the relationship between sense of presence and post-intervention state anxiety in the VR group, controlled for pre-intervention state anxiety. A significant negative association was observed (β = –0.528, p = 0.009), suggesting that a greater sense of presence was uniquely associated with reduced state anxiety following the VR intervention. State anxiety measured with STAI-X1 [46, 47], sense of presence measured with the IPQ [45]. All scores were z-standardized.

A multiple regression was conducted in the VR group to assess whether sense of presence measured with the IPQ predicted post-intervention anxiety, controlling for baseline anxiety (see Fig. 3). The model was significant (F(2, 18) = 11.08, p < 0.001), explaining 50.19% of the variance in post-treatment anxiety (R² = 0.55, adjusted R² = 0.50). Both predictors contributed meaningfully to the model. Pre-intervention anxiety was a significant positive predictor (β = 0.57, SE = 0.16, t(18) = 3.64, p = 0.002, 95% CI [0.24, 0.91]), while sense of presence was inversely related (β = −0.47, t(18) = −2.95, SE = 0.16, p = 0.009, 95% CI [−0.80, −0.13]).

## Discussion

This RCT systematically compared the feasibility of VR and GI relaxation in older adults with mental illness, as well as the effectiveness of these interventions in reducing negative emotions while increasing positive affect. Our findings support the feasibility of both relaxation methods in older age. Although dropout rates were slightly lower in the VR group, the difference was not significant. Participants in both conditions generally rated the intervention as satisfactory, immersive, helpful, and reported a sense of connectedness to nature, with scores similar to those observed in younger psychiatric populations [43].

Consistent with Just & Lütt et al. [32], demographic and clinical factors such as age, gender, main psychiatric diagnosis, psychiatric and somatic comorbidities, care level, and treatment setting showed no significant associations with feasibility or effectiveness outcomes. This suggests that both VR and GI relaxation may generally be suitable for older adults with mental illness, including very elderly individuals and those with serious mental health conditions.

Barriers appeared to differ between the interventions. In the GI group, reasons for dropout – including increased distress, anxiety, and difficulties with focus or imagination – aligned with previously reported limitations of GI [16, 28, 51]. Such issues were not reported for the VR intervention that possibly demanded less imaginative engagement, reducing barriers. Interestingly, the distressing memories reported as a dropout reason in the GI group were not reported in the VR group. This could indicate that this intervention, through technically supported immersion in predetermined scenarios, limits the cognitive drift into aversive situations. Regarding potential side effects of the VR intervention, motion sickness was minimal, echoing the findings of Just & Lütt et al. [32]. In line with this no participant discontinued due to motion sickness – contrasting with reports in younger samples [10]. The absence of other adverse events supports recent reviews [52], indicating that VR is generally well tolerated in older populations. However, we acknowledge that we did not examine all potential contraindications of GI and VR in older adults. For instance, visual impairments could present a relevant obstacle for VR relaxation and should be addressed in future research.

Both interventions produced significant immediate improvements in positive affect, relaxation, and well-being, as well as reductions in state anxiety, stress, and negative affect. Effect sizes ranged from η²p = 0.34–0.70, indicating large effects [53]. These findings align with previous studies reporting comparable pre-post improvements in anxiety and affect across similar approaches [10, 16, 28]. Large effects of the interventions suggest that they bear clinical relevance and should be considered in the treatment of older adults with mental illness.

Neither a main effect of intervention nor an interaction with time reached significance for any outcome. Unlike previous studies reporting greater effects of VR relaxation on affective outcomes [10, 54], we found no superiority of VR over GI in reducing negative affect or enhancing positive affect, stress, or relaxation. This discrepancy may be due to methodological differences, such as the use of multiple sessions, the design of VR content, or inclusion of a passive control group – factors absent in our single-session design with a treatment-as-usual control group. Furthermore, our trial may have lacked sufficient statistical power. Post-hoc power analyses for interaction effects showed adequate test power (≥ 0.75) for positive affect, stress, and well-being, but insufficient power for anxiety, relaxation, and negative affect. Thus, non-significant group differences, particularly on these outcomes, should be interpreted with caution, as meaningful intervention effects may not have been detected. Future studies should replicate these findings with larger samples and repeated sessions. Nevertheless, our results are consistent with other studies showing no differential effects of VR versus standard relaxation on anxiety, affect, or relaxation [16, 28, 55], and add to the growing evidence of VR’s therapeutic potential across psychiatric populations [25, 26, 29].

An intriguing result emerged regarding the role of presence in VR. The average sense of presence during VR relaxation was comparable to levels reported by Just & Lütt et al. in geriatric psychiatric inpatients [32] and considered high in other studies [56, 57]. Our exploratory regression analysis showed that presence significantly predicted post-treatment anxiety, even after controlling for baseline levels of anxiety. While higher initial anxiety predicted higher post-intervention anxiety, a stronger sense of presence was linked to greater symptom reduction. This aligns with earlier findings suggesting that presence facilitates anxiety reduction in VR relaxation [28, 55, 58], and it complements similar results for immersion [59, 60], a related construct [61]. These findings suggest that presence may influence therapeutic outcomes of VR interventions in geriatric psychiatry. Other research indicates that presence may also influence the effectiveness of other VR-based treatments for anxiety in older populations, where presence has shown both beneficial (relaxation) and mixed (exposure) effects [62].

Regarding further practical implementation of VR relaxation in geriatric psychiatry, we recommend conducting multi-session studies with longer follow-up periods. When determining the number of sessions, future research should account for the clinical setting and participants’ capabilities: outpatients may be able to use VR independently at home across multiple sessions, whereas inpatients with a high disease burden may require more support. Moreover, integrating VR into treatment plans should be accompanied by an economic evaluation of its costs and benefits. While VR may require less therapeutic guidance than GI, initial equipment costs and the potential need for technical support must be considered, especially in clinical settings with limited resources.

Several limitations of our trial should be noted. Due to limited prior research in older adults with mental illness, no reliable a priori sample size calculation was possible. While group allocation was automated, the assessor was not blinded during data collection and analyses, potentially introducing some bias. Feasibility and effectiveness were assessed via self-report, which can be prone to bias. We used single-item VAS, which have been proven to be psychometrically comparable to validated questionnaires for stress and well-being [63, 64], to reduce effort and burden for patients. However, this may have limited reliability of measurements [65]. Future research should incorporate validated multi-item scales in addition to single-item VAS to allow for validation and physiological indicators of relaxation such as heart rate or blood pressure. Despite randomization, the dependency on care differed between the two groups. Sensitivity analyses revealed no significant association between care dependency and effectiveness or feasibility. Nevertheless, this covariate imbalance might still have influenced our results and should be controlled for in future research. Furthermore, the heterogeneous sample does not allow conclusions about the differential effectiveness of VR across specific diagnostic groups, which should be examined in future studies. Although assessments were conducted within a single session and patients were included at least one week after a setting change, the influence of treatment adjustments on our results cannot be fully ruled out. Finally, only one relaxation session was conducted without follow-up measurements, and no passive control group was included, limiting insights into causality and long-term effects of the intervention and highlighting the need for future studies with repeated sessions and follow-up assessments.

## Conclusion

Our RCT suggests that VR-based relaxation is a feasible and effective alternative to GI for older adults with mental illness. Our findings highlight the comparable benefits of GI and VR relaxation, with significant short-term improvements and no notable differences in outcomes. Participants generally described both interventions as engaging and beneficial. The VR intervention was well tolerated, with minimal motion sickness and no adverse events, even among participants of advanced age or with serious mental illness. In aging societies with workforce shortages, VR offers a scalable option that patients can use independently. Further research is needed to validate these findings, identify contraindications, and assess real-world clinical integration. Still, our results support the inclusion of digital therapies in mental health care for older adults to promote equitable access to effective treatments and to ultimately reduce the ‘digital gray divide’ between different age groups with mental health conditions.

## Supplementary information

Appendix

## Data Availability

The data was used to generate results of this study are available from the corresponding author upon reasonable request.
