# Supplementary material for: Managing emotional distress in older adults with mental illness: a randomized controlled trial evaluating virtual reality relaxation
Source: Transl Psychiatry. 2026 Mar 20;16:162. doi: 10.1038/s41398-026-03955-1 (PMC13018184; doi:10.1038/s41398-026-03955-1)
Supplement: Supplementary file 1 — Appendix [file 41398_2026_3955_MOESM1_ESM.docx]

# **APPENDIX**

### **Appendix A: Script of the guided dream journey**

Finden Sie eine Position, um bequem zu sitzen. Schließen Sie sanft Ihre Augen und atmen Sie tief ein… – … und wieder aus. Spüren Sie, wie jeder Atemzug Sie mehr in den gegenwärtigen Moment bringt. – Lassen Sie Ihre Schultern immer mehr sinken und sich ganz entspannen. –

Lassen Sie alle Gedanken los, die Sie mitgebracht haben. – In diesem Moment sind Sie ganz bei sich. – Sie sind hier, um sich zu entspannen und sich selbst etwas Gutes zu tun. –

Stellen Sie sich nun vor, Sie gehen auf eine Reise zu einem wunderschönen, friedlichen Ort. – Sie befinden sich auf einer grünen Wiese. – Die Sonne scheint sanft und warm auf Ihre Haut. – Der Frühling hat die Erde erweckt, und Sie können das frische, grüne Gras um sich herum sehen. – Der Boden unter Ihnen ist weich, Sie nehmen den angenehmen Geruch von Moos wahr. – Die Luft ist klar und angenehm – der Duft von Blumen liegt in der Luft. –

Direkt vor Ihnen plätschert ein Fluss ruhig und gleichmäßig. – Sie hören das sanfte Rauschen des Wassers, das Ihnen ein Gefühl von Geborgenheit und Ruhe vermittelt. – Der Fluss zieht sich ruhig und friedlich durch die Wiese, und das Sonnenlicht glitzert auf der Wasseroberfläche. – Ein sanfter, lauer Wind streicht über Ihre Haut. –

Schauen Sie sich um und nehmen Sie die Details dieser friedlichen Szene wahr. – Der Himmel über Ihnen ist weit und klar. – Am Horizont erheben sich die Bäume eines Waldes, dunkelgrüne Tannen und Buchen wiegen sich leicht im Wind. – Sie hören die rauschenden Blätter der Bäume. – Dahinter sehen Sie majestätische Berge, ihre schneebedeckten Gipfel leuchten in der Sonne. –

Links von Ihnen entdecken Sie einen kleinen Hasen, der in der Wiese am Flussrand sitzt. – Der Hase bewegt sich vorsichtig und mit einer sanften Eleganz. – Seine Ohren wackeln leicht, und er blickt ruhig in Ihre Richtung. –

In der Mitte der Wiese, fast wie ein stiller Beobachter, sehen Sie ein Reh. – Es steht ganz ruhig. – Dann bewegt es sich mit einer anmutigen Leichtigkeit, als ob es ein Teil des Windes und der Natur selbst ist. – Sie spüren, wie der Moment in dieser friedlichen Umgebung langsamer verläuft und Sie sich vollkommen in der Stille und Harmonie dieser Landschaft verlieren können. –

Spüren Sie die Ruhe und den Frieden dieses Ortes. – Genießen Sie die Schönheit der Landschaft. – Nehmen Sie das Rauschen des Flusses wahr, den Blumenduft der Wiese, die Erhabenheit des Waldes und der Berge in der Ferne, die Wärme der Sonne. –

Spüren Sie die harmonische Ruhe der Tiere, die Sie umgeben. – Sie gehören zu dieser Landschaft, zu dieser Ruhe und Harmonie. –

Wenn Sie bereit sind, können Sie sich nun langsam von der Wiese, dem Fluss, dem Wald und den Tieren verabschieden. – Nehmen Sie Entspannung mit, die Sie in diesem Moment aufgenommen haben. –

Mit jedem Atemzug kommen Sie ein Stück mehr zurück in den Raum, in dem Sie sich jetzt befinden. – Wenn Sie nun bereit sind, öffnen Sie langsam Ihre Augen/nehmen Sie langsam die Brille ab, atmen Sie tief ein und kehren Sie mit einem Gefühl von Entspannung in den gegenwärtigen Moment zurück. –

*Note.* The first and last paragraphs served as the introduction and conclusion in both groups.

### **Appendix B: Scattered boxplots of patients’ experience and satisfaction by group.**

### **Figure B1:** Scattered boxplots comparing (A) patients’ experience and (B) satisfaction between VR and GI.

**
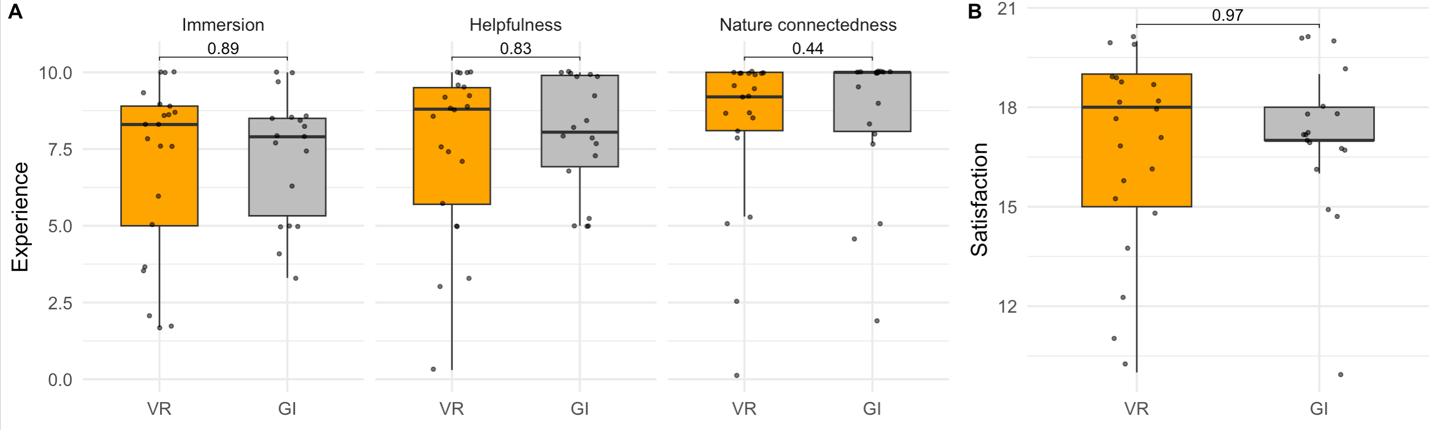
**

*Note.* Patients’ experience was measured with three VAS items (0 = not at all, 10 = very) and satisfaction with five ZUF-8 items. Mann-Whitney U test results between VR and GI are indicated by the bars above the plots (all p > 0.05).

### **Appendix C: Associations between sociodemographic/clinical variables and feasibility outcomes by group.**

| **Table C1.** Spearman correlations between sociodemographic/clinical variables and feasibility outcomes by group. | | | | | |
| --- | --- | --- | --- | --- | --- |
|  |  | **VR** | | **GI** | |
|  |  | Age | Education years | Age | Education years |
| Satisfaction | *r_S_* | -0.319 | -0.271 | -0.159 | -0.565 |
|  | *p FDR adj. p* | 0.159 0.413 | 0.235 0.522 | 0.529 0.661 | 0.015  0.147 |
| Immersion | *r_S_* | -0.208 | -0.315 | 0.029 | -0.193 |
|  | *p FDR adj. p* | 0.365  0.562 | 0.165  0.413 | 0.909  0.957 | 0.442  0.589 |
| Helpfulness | *r_S_* | -0.496 | -0.212 | 0.232 | -0.383 |
|  | *p*  *FDR adj. p* | 0.022  0.147 | 0.356  0.562 | 0.354  0.562 | 0.116  0.413 |
| Connectedness to nature | *r_S_* | -0.132 | -0.545 | -0.012 | -0.345 |
|  | *p*  *FDR adj. p* | 0.568  0.668 | 0.011  0.147 | 0.962  0.962 | 0.161  0.413 |
| Motion sickness | *r_S_* | 0.247 | 0.053 |  |  |
|  | *p*  *FDR adj. p* | 0.280  0.560 | 0.818  0.909 |  |  |
| IPQ mean | *r_S_* | -0.186 | -0.394 |  |  |
|  | *p*  *FDR adj. p* | 0.419  0.589 | 0.077  0.385 |  |  |
| *Note.* FDR Benjamini-Hochberg correction of p-values was conducted for 20 correlational analyses. | | | | | |

| **Table C2.** Mann-Whitney U-tests for comparing feasibility outcomes in sociodemographic/ clinical variables by group. | | | | | | | |
| --- | --- | --- | --- | --- | --- | --- | --- |
|  |  | **VR** | | | | | |
|  |  | Satisfaction | Immersion | Helpfulness | Connectedness to nature | Motion sickness | IPQ mean |
| Gender | *U* | 28.50 | 41.50 | 39.50 | 46.50 | 45.00 | 46.50 |
|  | *p*  *FDR adj. p* | 0.128  0.856 | 0.585  0.856 | 0.488  0.856 | 0.856  0.856 | 0.799  0.856 | 0.856  0.856 |
| Psychiatric comorbidity | *U* | 33.50 | 30.00 | 42.00 | 41.50 | 33.00 | 37.50 |
|  | *p*  *FDR adj. p* | 0.381  0.856 | 0.267  0.856 | 0.850  0.856 | 0.791  0.856 | 0.381  0.856 | 0.569  0.856 |
| Somatic comorbidity | *U* | 40.00 | 33.50 | 40.00 | 34.00 | 33.00 | 34.50 |
|  | *p*  *FDR adj. p* | 0.733  0.856 | 0.381  0.856 | 0.733  0.856 | 0.424  0.856 | 0.381  0.856 | 0.424  0.856 |
|  | **GI** | | | | | | |
| Gender | *U* | 18.00 | 25.00 | 7.00 | 32.00 |  |  |
|  | *p*  *FDR adj. p* | 0.055  0.825 | 0.203  0.856 | 0.002  0.06 | 0.515  0.856 |  |  |
| Psychiatric comorbidity | *U* | 36.00 | 36.00 | 32.00 | 36.00 |  |  |
|  | *p*  *FDR adj. p* | 0.762  0.856 | 0.762  0.856 | 0.515  0.856 | 0.762  0.856 |  |  |
| Somatic comorbidity | *U* | 29.50 | 30.50 | 21.50 | 29.50 |  |  |
|  | *p*  *FDR adj. p* | 0.775  0.856 | 0.849  0.856 | 0.289  0.856 | 0.775  0.856 |  |  |
| *Note.* FDR Benjamini-Hochberg correction of p-values was conducted for 30 comparisons. | | | | | | | |

| **Table C3.** Kruskal-Wallis tests for comparing feasibility outcomes in sociodemographic/ clinical variables by group. | | | | | | | |
| --- | --- | --- | --- | --- | --- | --- | --- |
|  |  | **VR** | | | | | |
|  |  | Satisfaction | Immersion | Helpfulness | Connectedness to nature | Motion sickness | IPQ mean |
| Psychiatric diagnosis | *H* | 6.17 | 3.62 | 6.27 | 4.92 | 3.48 | 5.47 |
|  | *p* | 0.187 | 0.460 | 0.180 | 0.296 | 0.481 | 0.243 |
| Setting | *H* | 6.17 | 3.62 | 6.27 | 4.92 | 3.48 | 5.47 |
|  | *p* | 0.187 | 0.460 | 0.180 | 0.296 | 0.481 | 0.243 |
| Care level | *H* | 1.57 | 0.51 | 3.03 | 0.68 | 0.39 | 0.46 |
|  | *p* | 0.455 | 0.776 | 0.220 | 0.713 | 0.823 | 0.796 |
|  |  | **GI** | | | | | |
| Psychiatric diagnosis | *H* | 2.06 | 2.42 | 3.92 | 1.28 |  |  |
|  | *p* | 0.725 | 0.660 | 0.418 | 0.864 |  |  |
| Setting | *H* | 0.32 | 0.64 | 3.71 | 0.05 |  |  |
|  | *p* | 0.854 | 0.726 | 0.156 | 0.974 |  |  |
| Care level | *H* | 4.26 | 2.97 | 7.67 | 0.66 |  |  |
|  | *p* | 0.235 | 0.396 | 0.053 | 0.882 |  |  |
| *Note.* No correction for multiple testing was conducted as none of the differences were significant. | | | | | | | |

### **Appendix D: Group comparisons at baseline (T0) for all effectiveness outcome variables.**

| **Table D1.** Independent t-tests for comparing values in outcome variables at baseline (T0) between participants in condition VR vs. GI. | | | | | | | |
| --- | --- | --- | --- | --- | --- | --- | --- |
|  | ***t*** | **df** | ***p*** | **Mean Difference** | **Std. Error Difference** | **Lower 95% CI** | **Upper 95% CI** |
| State anxiety | 0.12 | 37 | 0.909 | 0.37 | 3.22 | -6.16 | 6.91 |
| Positive affect | -0.43 | 37 | 0.668 | -0.97 | 2.24 | -5.51 | 3.57 |
| Negative affect | -0.10 | 37 | 0.923 | -0.17 | 1.71 | -3.64 | 3.31 |
| Stress | 1.68 | 37 | 0.101 | 13.83 | 8.24 | -2.85 | 30.52 |
| Relaxation | -0.30 | 37 | 0.766 | -2.60 | 8.68 | -20.19 | 14.98 |
| Well-being | -0.61 | 37 | 0.547 | -5.17 | 8.49 | -22.37 | 12.04 |
| *Note.* No correction for multiple testing was conducted as none of the differences were significant. | | | | | | | |

### **Appendix E: Bootstrapped ANOVAs confirming the robustness of the time effects.**

**Table E1.** Bootstrapped test statistics for negative affect and stress.

|  | ***Effect*** | **df** | ***F*** | **SD** | **95% CI (F)** | **η²p** | **95% CI (η²p)** |
| --- | --- | --- | --- | --- | --- | --- | --- |
| Negative affect | Time | 1,37 | 14.36 | 2.94 | [9.21, 20.32] | 0.34 | [0.27, 0.43] |
|  | Group | 1,37 | 0.42 | 0.84 | [0.00, 2.70] | 0.01 | [0.00, 0.09] |
|  | Time x Group | 1,37 | 0.29 | 0.49 | [0.00, 1.83] | 0.01 | [0.00, 0.07] |
| Stress | Time | 1,37 | 43.12 | 6.65 | [30.95, 56.65] | 0.61 | [0.55, 0.68] |
|  | Group | 1,37 | 2.48 | 1.73 | [0.16, 6.79] | 0.08 | [0.01, 0.21] |
|  | Time x Group | 1,37 | 1.34 | 1.14 | [0.01, 4.18] | 0.05 | [0.00, 0.14] |
| *Note.* T = Time; G = Group; F-values represent the mean of the bootstrapped distribution; SD =  standard deviation of F across 1000 resamples; 95% CI(F) = 95% confidence interval of the  bootstrapped F statistic. | | | | | | | |

### **Appendix F: Post-hoc pairwise comparisons of the ANOVA.**

**Table F1.** Post-hoc pairwise comparisons (paired t-tests) of the ANOVA main effects of time.

| **Outcome** | **Group** | **MD**  **[95% CI]** | **SE** | **t** | **p-value** | **FDR adj. p-value** | **dz**  **[95% CI]** |
| --- | --- | --- | --- | --- | --- | --- | --- |
| State Anxiety | VR | -12.48  [-16.56, -8.40] | 1.96 | 6.38 | < 0.001 | < 0.001 | -0.99  [-1.61, -0.52] |
|  | GI | -11.11  [-14.48, -7.74] | 1.60 | 6.95 | < 0.001 | < 0.001 | -1.13  [-1.92, -0.58] |
| Positive affect | VR | 6.62  [4.09, 9.15] | 1.21 | -5.45 | < 0.001 | < 0.001 | 0.70  [0.22, 1.25] |
|  | GI | 5.22  [2.65, 7.79] | 1.22 | -4.29 | < 0.001 | < 0.001 | 0.59  [0.10, 1.21] |
| Negative affect | VR | -3.48  [-5.89, -1.06] | 1.16 | 3.01 | 0.007 | 0.007 | -0.58  [-0.96, -0.26] |
|  | GI | -3.39  [-5.61, -1.16] | 1.06 | 3.21 | 0.005 | 0.0054 | -0.74  [-1.19, -0.31] |
| Relaxation | VR | 3.40  [2.23, 4.57] | 0.56 | -6.09 | < 0.001 | < 0.001 | 1.26  [0.64, 1.98] |
|  | GI | 3.26  [1.92, 4.61] | 0.64 | -5.12 | < 0.001 | < 0.001 | 1.04  [0.58, 1.66] |
| Well-being | VR | 3.22  [2.26, 4.19] | 0.46 | -6.96 | < 0.001 | < 0.001 | 1.05  [0.61, 1.61] |
|  | GI | 2.25  [1.35, 3.15] | 0.43 | -5.28 | < 0.001 | < 0.001 | 0.79  [0.29, 1.43] |
| Stress | VR | -3.30  [-4.45, -2.15] | 0.55 | 5.97 | < 0.001 | < 0.001 | -1.09  [-1.68, -0.59] |
|  | GI | -2.41  [-3.46, -1.35] | 0.50 | 4.81 | < 0.001 | < 0.001 | -1.01  [-1.56, -0.54] |

*Note.* MD = mean difference (T1 - T0); SE = standard error; 95% CI = 95% confidence intervals; adjusted p-values based on FDR Benjamini-Hochberg correction for 12 tests; dz = Cohen’s d for paired data. State anxiety measured with STAI-X1 [[46,47]](https://www.zotero.org/google-docs/?XM9BAN), positive/negative affect measured with PANAS [[49,50]](https://www.zotero.org/google-docs/?1ilQKi), single-item VAS for stress (0 = "no stress" to 10 = "extreme stress"), relaxation (0 = "not relaxed at all" to 10 = "very relaxed"), and well-being (0 = "not well at all" to 10 = "very well").

#

### **Appendix G: Associations between sociodemographic/clinical variables and change in effectiveness outcomes by group.**

| **Table G1.** Spearman correlations between sociodemographic/clinical variables and difference scores of effectiveness outcomes by group. | | | | | |
| --- | --- | --- | --- | --- | --- |
|  |  | **VR** | | **GI** | |
|  |  | Age | Education years | Age | Education years |
| State anxiety | *r_S_* | -0.172 | 0.245 | 0.485 | 0.064 |
|  | *p FDR adj. p* | 0.456  0.730 | 0.285  0.589 | 0.041  0.271 | 0.802  0.922 |
| Positive affect | *r_S_* | -0.227 | -0.347 | -0.108 | -0.201 |
|  | *p FDR adj. p* | 0.321  0.593 | 0.123  0.368 | 0.670  0.893 | 0.423  0.726 |
| Negative affect | *r_S_* | -0.005 | 0.423 | 0.261 | 0.117 |
|  | *p*  *FDR adj. p* | 0.982  0.982 | 0.056  0.271 | 0.295  0.589 | 0.643  0.893 |
| Relaxation | *r_S_* | 0.139 | -0.383 | -0.399 | -0.062 |
|  | *p*  *FDR adj. p* | 0.549  0.824 | 0.086  0.345 | 0.101  0.345 | 0.806  0.922 |
| Well-being | *r_S_* | 0.078 | -0.422 | -0.265 | 0.009 |
|  | *p*  *FDR adj. p* | 0.738  0.922 | 0.056  0.271 | 0.288  0.589 | 0.972  0.982 |
| Stress | *r_S_* | 0.033 | 0.317 | 0.473 | 0.535 |
|  | *p*  *FDR adj. p* | 0.886  0.967 | 0.162  0.432 | 0.048  0.271 | 0.022  0.271 |
| *Note.* FDR Benjamini-Hochberg correction of p-values was conducted for 24 correlational analyses. | | | | | |

| **Table G2.** Mann-Whitney U-tests for comparing difference scores of effectiveness outcomes in sociodemographic/ clinical variables by group. | | | | | | | |
| --- | --- | --- | --- | --- | --- | --- | --- |
|  |  | **VR** | | | | | |
|  |  | State anxiety | Positive affect | Negative affect | Relaxation | Well-  being | Stress |
| Gender | *U* | 42.50 | 44.00 | 44.50 | 44.50 | 35.00 | 35.50 |
|  | *p* | 0.636 | 0.743 | 0.743 | 0.743 | 0.322 | 0.322 |
| Psychiatric comorbidity | *U* | 30.00 | 39.00 | 43.00 | 42.50 | 37.50 | 44.00 |
|  | *p* | 0.267 | 0.677 | 0.910 | 0.850 | 0.569 | 0.970 |
| Somatic comorbidity | *U* | 21.50 | 42.00 | 27.50 | 31.50 | 24.50 | 37.00 |
|  | *p* | 0.066 | 0.850 | 0.178 | 0.302 | 0.112 | 0.569 |
|  | **GI** | | | | | | |
| Gender | *U* | 33.50 | 36.50 | 34.50 | 31.50 | 30.50 | 26.00 |
|  | *p* | 0.573 | 0.762 | 0.633 | 0.460 | 0.408 | 0.237 |
| Psychiatric comorbidity | *U* | 31.50 | 27.50 | 33.50 | 29.50 | 37.50 | 34.00 |
|  | *p* | 0.460 | 0.274 | 0.573 | 0.360 | 0.829 | 0.633 |
| Somatic comorbidity | *U* | 29.00 | 18.50 | 28.50 | 19.00 | 27.50 | 21.00 |
|  | *p* | 0.775 | 0.173 | 0.703 | 0.208 | 0.633 | 0.289 |
| *Note.* No correction for multiple testing was conducted as none of the differences were significant. | | | | | | | |

| **Table G3.** Kruskal-Wallis tests for comparing difference scores of effectiveness outcomes in sociodemographic/ clinical variables by group. | | | | | | | |
| --- | --- | --- | --- | --- | --- | --- | --- |
|  |  | **VR** | | | | | |
|  |  | State anxiety | Positive affect | Negative affect | Relaxation | Well-  being | Stress |
| Psychiatric diagnosis | *H* | 0.45 | 4.59 | 2.75 | 3.13 | 1.37 | 5.51 |
|  | *p*  *FDR adj. p* | 0.978  0.990 | 0.332  0.648 | 0.601  0.832 | 0.536  0.804 | 0.849  0.956 | 0.239  0.648 |
| Setting | *H* | 1.63 | 0.15 | 2.21 | 2.32 | 3.27 | 2.29 |
|  | *p*  *FDR adj. p* | 0.443  0.759 | 0.929  0.984 | 0.331  0.648 | 0.313  0.648 | 0.195  0.648 | 0.318  0.648 |
| Care level | *H* | 1.33 | 0.83 | 2.15 | 2.71 | 2.27 | 2.70 |
|  | *p*  *FDR adj. p* | 0.515  0.804 | 0.660  0.880 | 0.342  0.648 | 0.258  0.648 | 0.321  0.648 | 0.260  0.648 |
|  |  | **GI** | | | | | |
| Psychiatric diagnosis | *H* | 7.76 | 7.28 | 6.20 | 5.30 | 1.24 | 5.06 |
|  | *p*  *FDR adj. p* | 0.101  0.648 | 0.122  0.648 | 0.185  0.648 | 0.258  0.648 | 0.872  0.972 | 0.281  0.648 |
| Setting | *H* | 4.96 | 5.24 | 10.75 | 2.49 | 0.74 | 1.17 |
|  | *p*  *FDR adj. p* | 0.084  0.648 | 0.073  0.648 | 0.005  0.180 | 0.289  0.648 | 0.693  0.891 | 0.558  0.804 |
| Care level | *H* | 0.90 | 0.69 | 2.88 | 1.25 | 0.11 | 2.45 |
|  | *p*  *FDR adj. p* | 0.825  0.956 | 0.876  0.956 | 0.410  0.738 | 0.742  0.921 | 0.990  0.990 | 0.484  0.792 |
| *Note.* FDR Benjamini-Hochberg correction of p-values was conducted for 36 comparisons. | | | | | | | |
